# Identification of Associations between Bacterioplankton and Photosynthetic Picoeukaryotes in Coastal Waters

**DOI:** 10.3389/fmicb.2016.00339

**Published:** 2016-03-22

**Authors:** Hanna M. Farnelid, Kendra A. Turk-Kubo, Jonathan P. Zehr

**Affiliations:** ^1^Ocean Sciences Department, University of California at Santa CruzSanta Cruz, CA, USA; ^2^Centre for Ecology and Evolution in Microbial Model Systems, Linnaeus UniversityKalmar, Sweden

**Keywords:** microbial associations, bacterivory, flow cytometry, photosynthetic picoeukaryotes, symbiosis

## Abstract

Photosynthetic picoeukaryotes are significant contributors to marine primary productivity. Associations between marine bacterioplankton and picoeukaryotes frequently occur and can have large biogeochemical impacts. We used flow cytometry to sort cells from seawater to identify non-eukaryotic phylotypes that are associated with photosynthetic picoeukaryotes. Samples were collected at the Santa Cruz wharf on Monterey Bay, CA, USA during summer and fall, 2014. The phylogeny of associated microbes was assessed through 16S rRNA gene amplicon clone and Illumina MiSeq libraries. The most frequently detected bacterioplankton phyla within the photosynthetic picoeukaryote sorts were Proteobacteria (*Alphaproteobacteria* and *Gammaproteobacteria*) and Bacteroidetes. Intriguingly, the presence of free-living bacterial genera in the photosynthetic picoeukaryote sorts could suggest that some of the photosynthetic picoeukaryotes were mixotrophs. However, the occurrence of bacterial sequences, which were not prevalent in the corresponding bulk seawater samples, indicates that there was also a selection for specific OTUs in association with photosynthetic picoeukaryotes suggesting specific functional associations. The results show that diverse bacterial phylotypes are found in association with photosynthetic picoeukaryotes. Taxonomic identification of these associations is a prerequisite for further characterizing and to elucidate their metabolic pathways and ecological functions.

## Introduction

Marine ecosystems are comprised of networks of interacting organisms and marine microbes that are frequently associated (Anderson, 2014; Cooper and Smith, 2015). Interestingly, associated bacteria are often distinct from species or strains in the surrounding seawater (Goecke et al., 2013) suggesting that there is niche speciation and co-evolution in habitat-specific cell consortia. Phytoplankton–bacteria associations can range from commensal to mutualistic or parasitic, and bacterial associations are known to directly influence phytoplankton growth, reproduction, and mortality (Grossart and Simon, 2007; Sher et al., 2011). Bacteria may also colonize dying or dead phytoplankton or protists which provide a source of organic matter (Azam and Malfatti, 2007). Thereby, associations between marine microorganisms play an important role in the food web and can have large impacts on phytoplankton dynamics and biogeochemical cycling.

In the marine environment, the majority of phytoplankton cells are microscopic and unicellular. Consequently bacteria–phytoplankton associations are methodologically challenging to study. The phycosphere is a region in close proximity to phytoplankton cells assumed to be rich in organic matter that attracts free-living bacteria (Stocker, 2012). Although species-specific bacteria–phytoplankton associations have been described it remains difficult to determine the metabolic functions and exchange between associated cells (Sapp et al., 2007; Worden et al., 2015). Recently, single cell sorting has been used to identify associations between bacteria and individual diatom cells and picoeukaryotes (Yoon et al., 2011; Martinez-Garcia et al., 2012a; Baker and Kemp, 2014), providing increasing evidence of close cell-to-cell associations in marine ecosystems. In addition, links between the phylogeny and metabolic function of an organism may be identified (e.g., Stepanauskas and Sieracki, 2007; Swan et al., 2011) which will be important for characterizing bacteria–phytoplankton interactions.

Photosynthetic picoeukaryotes are significant contributors to phytoplankton biomass and primary production (Worden et al., 2004; Jardillier et al., 2010). Interestingly, an increasing number of studies have demonstrated that photosynthetic picoeukaryotes can be mixotrophs (González et al., 1993; Zubkov and Tarran, 2008; Sanders and Gast, 2012; Hartmann et al., 2013; McKie-Krisberg and Sanders, 2014). With such trophic flexibility, photosynthetic picoeukaryotes have the potential to dominate both primary production and control bacterioplankton abundances in diverse marine ecosystems (Hartmann et al., 2012; McKie-Krisberg and Sanders, 2014). The recent discovery of a symbiosis between a photosynthetic picoeukaryote and the nitrogen-fixing cyanobacterium *Atelocyanobacterium thalassa* (UCYN-A; Thompson et al., 2012), contributing significantly to global nitrogen fixation (Montoya et al., 2004), suggests that associations with photosynthetic picoeukaryotes may also be important in key biogeochemical processes.

In this study, we aimed to determine if there were associations between bacterioplankton and photosynthetic picoeukaryotes. Seawater samples were collected from the Santa Cruz wharf on Monterey Bay, CA, USA, a coastal site with high photosynthetic picoeukaryote abundances. Using flow cytometry, populations of photosynthetic picoeukaryotes were sorted and analyzed. The associated bacterioplankton cells were characterized using 16S rRNA gene amplicon clone and Illumina MiSeq libraries. In addition, bacterial isolates from photosynthetic picoeukaryote populations were obtained, providing potential model systems and gene targets for future studies. The results describe the occurrence patterns and phylogenetic specificity of associated cells and provide knowledge of species associations forming the foundation of the marine ecosystem.

## Materials and Methods

### Sampling

Surface seawater was collected in the morning from the Santa Cruz wharf (36.95 N, 122.02 W) in Monterey Bay, CA in summer and fall, 2014 using a 10 L bucket. Water was transferred into an acid washed sampling bottle, and transported immediately to University of California Santa Cruz (UCSC) for processing. As part of the Monterey Bay weekly phytoplankton sampling program, samples for phytoplankton community assessment and nutrient analyses were collected. Net plankton samples were collected with a 20 μm mesh net in the upper 3 m of the water column. The live net tow material was viewed under a dissecting microscope at 64× magnification. The Relative Abundance Index (RAI) of the most frequently observed genera of dinoflagellates and diatoms were recorded according to the system of the California Department of Public Health (CDPH) Phytoplankton Monitoring Program (Jester et al., 2009). For nutrient analyses, water was filtered onto 0.7 μm GF/F filters (Whatman, GE Healthcare, Little Chalfont, UK), placed into Falcon centrifuge tubes and stored at –20°C until processing. Nitrate, phosphate, and silicate were analyzed using a Lachat QuikChem 8500 Flow Injection Analyst System and Omnion 3.0 software (Lachat Instruments; Hach Company, Loveland, CO, USA). Ammonium and urea were analyzed fluorometrically as described by Gibble and Kudela (2014).

For DNA samples of bulk seawater, 300–500 ml seawater was pre-filtered through a 10 μm polycarbonate filter and subsequently though a 0.2 μm supor filter (Pall Corporation, New York, NY, USA) in 25 mm Swinnex filter holders (Millipore, Billerca, MA, USA) using a peristaltic pump. The filters were placed in sterile 1.5 ml cryovials containing 0.1 g autoclaved glass beads, flash frozen in liquid nitrogen and stored at –20°C until DNA extraction. Seawater samples to be sorted for catalyzed reporter deposition – fluorescence *in situ* hybridization (CARD-FISH) were collected, as described above, in fall 2015 (September 15 and October 27), but no complementary phytoplankton, nutrient or DNA samples were collected at this time.

### DNA Extraction

Community DNA was extracted using the Qiagen Plant Minikit and QIAcube (Qiagen, Valencia, CA, USA). Briefly, 400 μl AP1 buffer (Qiagen Plant Minikit) was added to the sample tubes, followed by three freeze-thaw cycles using liquid nitrogen and a 65°C water bath (30 s and 2 min, respectively). The tubes were placed in a FastPrep-24 bead beater (MP Biomedicals, Irvine, CA, USA) and shaken at full speed for 2 min. The samples were Proteinase K treated for 1 h at 55°C with moderate shaking using 45 μl of Proteinase K (20 mg ml^-1^; Qiagen) and treated with 4 μl RNase A and incubated for 10 min at 65°C. The filters were removed using sterile needles, 130 μl AP2 buffer (Qiagen Plant Minikit) was added to each tube, and samples were then vortexed and incubated on ice for 10 min. To pellet the precipitates and beads, the tubes were centrifuged for 5 min at 14 000 rpm at 4°C, and the supernatant was transferred to 2 ml sample tubes and placed in the QIAcube for further extraction steps according to the manufacturer’s protocol. The samples were eluted using 100 μl AE buffer (Qiagen Plant Minikit) and concentrations were measured fluorometrically using Picogreen according to the manufacturer’s protocol. Extracted DNA samples were stored at –20°C.

### Flow Cytometry Sorting

**FIGURE 1:**
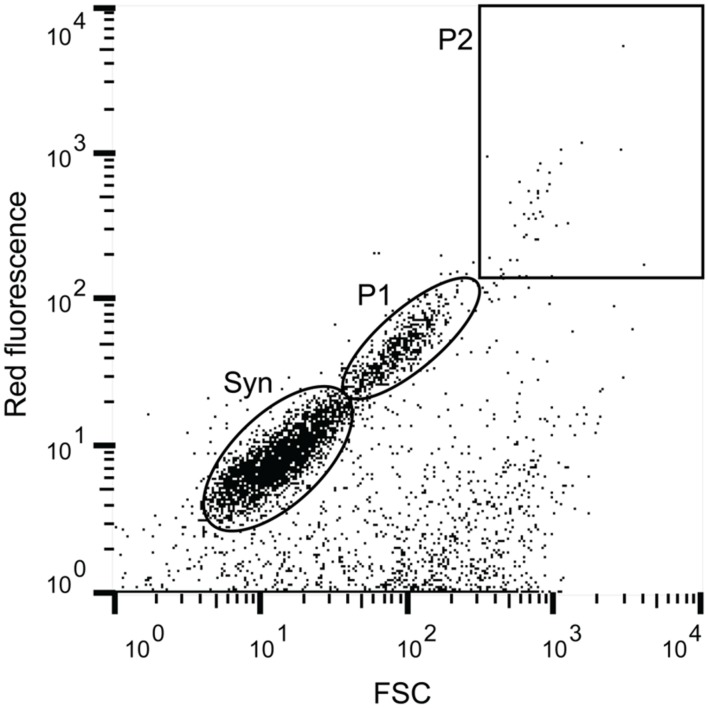
**Example of a flow cytogram with the cell populations that were targeted in this study**. Red fluorescence was used as a proxy for chlorophyll *a* content and forward scatter (FSC) as a proxy for size. The indicated cell populations are Syn (*Synechococcus*) as defined by phycoerythrin content (Orange fluorescence), and two size fractions of photosynthetic picoeukaryotes, small (P1, ∼ <3 μm beads) and large (P2, ∼ >3 μm beads).

Populations of photosynthetic picoeukaryotes were sorted using a BD Biosciences Influx Cell Sorter equipped with a small particle detector (BD Biosciences, San Jose, CA, USA) and a 488 nm Sapphire laser (Coherent, Santa Clara, CA, USA). The sorting was done using sterile filtered BioSure Sheath fluid diluted in ultrapure water to 1x concentration. Prior to flow cytometric sorting, the seawater samples were prefiltered using a 30 μm filter (Partec CellTrics, Swedesboro, NJ, USA) to prevent clogging of the 70 μm diameter nozzle. To minimize the risk of dislodging associations there was no concentration step and only fresh samples were processed. Data collection and sorting was triggered in the forward scatter (FSC) channel. The sorting gates were constructed in the BD FACS software using FSC as a proxy for cell size and red fluorescence (692–740 nm) as a proxy for chlorophyll *a* content. Photosynthetic picoeukaryotes were distinguished from *Synechococcus* populations using a NOT gate, deselecting cells with orange fluorescence (572–627 nm; i.e., phycoerythrin containing cells). For photosynthetic picoeukaryotes a smaller (P1, ∼ <3 μm beads) and a larger (P2, ∼ >3 μm beads) population was sorted (see **Figure 1** and Supplementary Figure 1). All sorts were collected in “1.0 Drop Purity” mode, and in combination with the small particle detection capabilities and triggering sorts on the FSC signal, this configuration is the optimal set-up for ensuring a low probability of co-sorting of unattached particles. Cell counts and cytograms were processed in FlowJo v10.0.7 (Tree Star). To minimize the risk of contamination, fluidic lines were regularly decontaminated with 1% bleach.

For sequencing, populations of 100–1,000 cells were sorted directly into sterile PCR tubes. To investigate if sheath fluid or the fluidic system of the cytometer was a source of contamination in the sort samples, sheath fluid was sampled using the test stream mode. For PCR applications, 2 μl aliquots of the sheath fluid, were collected on all but one sampling occasion, corresponding to the volume of approximately 2,000 sorted cells. Samples were stored at –80°C until further processing. To minimize cell loss, no additional DNA extraction step was added before PCR applications (Thompson et al., 2012). For CARD-FISH analyses, populations of 15,000 and 30,000 photosynthetic picoeukaryote cells (P1) were sorted into a PCR tube or onto a Poly-Prep slide (Sigma–Aldrich, St. Louis, MO, USA) and fixed in an equal volume of PBS-buffered 2% EM-grade paraformaldehyde (Electron Microscopy Sciences, Hatfield, PA, USA) for >1 h at room temperature in the dark. Poly-Prep slides were dried in a Bambino Hybridization Oven (Boekel Scientific, Feasterville-Trevose, PA, USA) at 28°C for ∼20 min and stored at –20°C until further processing. Samples in PCR tubes were frozen at –80°C until further processing.

### 16S rRNA Gene Clone Libraries

For all PCR applications extra precautions were taken to minimize contamination risks. Preparations took place in a UV hood in an amplicon free room using UV exposed tubes and 5 kD filtered ultrapure water. For clone libraries, a fragment of the 16S rRNA gene was amplified from sorted populations using the 27F (5′-AGA GTT TGA TCM TGG CTC AG-3′) or the 895F primer (5′-CRC CTG GGG AGT RCR G-3′) and 1492R (5′-GGT TAC CTT GTT ACG ACT T-3′). The 895F primer was previously designed to facilitate exploration of bacterial diversity in samples where DNA concentrations from cyanobacteria and plastids were high (Hodkinson and Lutzoni, 2010) and could thereby increase the proportion of non-Eukaryota sequences in the libraries. The composition of the 25 μl PCR mixture was 1x PCR buffer, 0.3 μl Invitrogen Platinum Taq (Invitrogen, Carlsbad, CA, USA), 2.5 mM MgCl_2_, 200 μM dNTP mix, and 0.5 μM of the respective primers. The PCR conditions were as follows: 3 min initial denaturation at 95°C, followed by 30 cycles of 30 s denaturation at 95°C, annealing for 30 s at 55°C, and elongation for 60 s at 72°C, and ending with a final elongation for 7 min at 72°C. Samples with sheath fluid and negative controls with only ultrapure water added to the reactions were run together with the sorted samples. Neither the sheath fluid controls nor the negative controls produced amplicons that could be visualized using gel electrophoresis (1.2% agarose, 1 h, 86 V), whereas sorted populations produced strong amplification products.

The amplified PCR products were purified (QIAquick PCR purification; Qiagen) and cloned using the Invitrogen TOPO TA cloning kit for sequencing according to the manufacturer’s protocol. Plasmid DNA was extracted in 96-well plates using the Montage Plasmid Miniprep Kit (EMD Millipore, Merck KGaA, Darmstadt, Germany) following manufacturer’s instructions. Sanger sequencing was done at the UC Berkeley DNA Sequencing Facility^1^. Low quality sequences were removed and the sequences were screened for chimeras using Decipher^2^. Sequences were deposited in GenBank under accession numbers KT906713–KT906977. For phylogenetic analyses of the sequences, clustering was done using CD-HIT-EST (Huang et al., 2010) with a 97% similarity level (Stackebrandt and Goebel, 1994). Sequences were taxonomically classified using the RDP classifier (Wang et al., 2007) and closest relatives were determined using NCBI Blastn. Neighbor-joining trees were constructed using MEGA6 using ClustalW for sequence alignment and neighbor-joining and maximum composite likelihood for distance estimation with 1,000 bootstrap replications (Tamura et al., 2013).

### Isolates

To isolate bacteria from photosynthetic picoeukaryote sorts on agar plates, cells (P1) from the October 22 sample were sorted onto Zobell agar plates (5 g peptone, 1 g yeast extract, 15 g bacto agar in 800 ml 0.2 μm filtered Santa Cruz wharf seawater and 200 ml ultrapure water). To assess if live bacteria were present in the sheath fluid, plates were also inoculated with 20 puddles of sheath fluid that had passed through the cytometer (∼10 μl each, corresponding to the volume of ∼10,000 sorted cells). The plates were incubated at room temperature in the dark for a total of 4 weeks. As colonies grew, they were clean streaked onto fresh Zobell agar plates three times prior to inoculation into 5 ml Zobell medium (5 g peptone, 1 g yeast extract in 800 ml, 0.2 μm filtered, Santa Cruz wharf seawater and 200 ml ultrapure water). DNA from the isolates was extracted using the E.Z.N.A Tissue DNA kit (Omega Biotek, Norcross, GA, USA) according to manufacturer’s protocol. 16S rRNA genes were amplified using the 27F and 1492R primers and the amplicons were bi-directionally sequenced. The sequence analyses were performed as described above and sequences were deposited in GenBank under accession numbers KT906978–KT907037.

### High-Throughput 16S rRNA Gene Amplicon Sequence Libraries

The associated bacteria within the sorted photosynthetic picoeukaryote populations and the microbial composition of the bulk seawater that the populations were sorted from were further investigated using next generation sequencing (NGS). The dominance of chloroplast sequences in the libraries from the sorted samples was less of a concern due to the depth of total sequencing. The PCR was performed in 25 μl reaction volumes as described above but with 0.25 μM of each primer. The PCR conditions were as follows: 5 min initial denaturation at 95°C, followed by 30 cycles of 40 s, denaturation at 95°C, annealing for 40 s at 53°C and elongation for 60 s at 72°C, and ending with a final elongation for 7 min at 72°C. When working with samples with low cell abundances it is important to take extra precaution against background contamination as it could have a large impact on the signal from the sample itself. NGS amplicon sequencing is highly sensitive and consequently low detection of microbial contaminants is an unavoidable and a consistent feature (e.g., Laurence et al., 2014; Salter et al., 2014). Although the two negative (water only) PCR controls were not visible using agarose gel electrophoresis, these were included in the pool of amplicons with unique separate barcodes.

The widely used primer set 341F and 806R, targeting the V3–V4 variable region of the 16S rRNA gene of Bacteria (Caporaso et al., 2011; Herlemann et al., 2011), was used for amplification, with slight modifications. Briefly, genomic DNA extracted from sorted populations was used as template for PCR amplification using a targeted amplicon sequencing (TAS) approach, as described by Bybee et al. (2011) and Green et al. (2015). In the first of the two-stage amplification procedure, the template was amplified (25 cycles) using gene-specific primers containing previously described CS1 and CS2 linkers (Moonsamy et al., 2013). The amplicons were then submitted for sequencing to the DNA Services (DNAS) Facility at the University of Illinois at Chicago. At the DNAS Facility, a second PCR amplification (eight cycles) was performed to incorporate barcodes and sequencing adapters into the final PCR products. These amplifications were performed using the Fluidigm Access Array barcoding system (Fluidigm, South San Francisco, CA, USA). Primers synthesized by Fluidigm contained Illumina sequencing adapters, sample-specific barcodes (10 bases, with a minimum hamming distance of 3), and CS1 and CS2 linkers (see Green et al., 2015). The large hamming distance minimizes the risk of mis-assignment of reads to the wrong sample which can occur at low frequencies in NGS datasets (e.g., Kircher et al., 2012). Sequencing was performed on an Illumina MiSeq sequencer at the DNAS facility using standard V3 chemistry with paired-end, 300 base reads. Fluidigm sequencing primers, targeting the CS1 and CS2 linker regions, were used to initiate sequencing. Demultiplexing of reads was performed on instrument.

The resulting paired-end FASTQ files were merged using the software package PEAR (Zhang et al., 2014). The software package CLC genomics workbench (v7; CLC Bio, Qiagen, Boston, MA, USA) was used for primer, quality (Q20) and length trimming (sequences <390 bp were removed). A chimera check (USEARCH61; Edgar et al., 2011) was performed and putative chimeras were removed from the dataset. Subsequently, sequences were clustered and taxonomy was assigned with the Greengenes database (v13_8) as reference using the QIIME bioinformatics pipeline (Caporaso et al., 2010). For phylogenetic analyses, operational taxonomic units (OTUs) were defined based on 97% similarity clustering using CD-HIT-EST with >10 sequence reads and performed as described above. Mitochondrial sequences and sequences affiliated with species not associated with environmental studies as closest relatives were identified and removed manually from the dataset. A Bray-Curtis similarity matrix was calculated based on OTUs normalized to the total number of sequences for each sample using the software package Primer6 (Primer-E). The sequences have been submitted to NCBI Sequence Read Archive (SRA) with accession number SRP065334.

### Microscopy

The presence of bacteria associated with picoeukaryotes was investigated visually using CARD-FISH as described by Pernthaler et al. (2002) with modifications described below. The probes used in this study were the general bacteria probe, EUB338 (Amann et al., 1990) and GAM42a (Manz et al., 1992), targeting *Gammaproteobacteria*. Unspecific binding and/or background autofluorescence was examined using the non-sense probe non338 (Wallner et al., 1993) and no probe controls. Pre-sorted fixed samples were thawed and pipetted onto pre-coated poly-L-lysine slides (Poly-Prep slides, Sigma–Aldrich) and dried in a Bambino Hybridization Oven (Boekel Scientific) at 28°C for ∼20 min. As an initial test of the protocol, 15 μl culture of isolates 1, 2, 12 and 15 (**Table 2**) were fixed and mounted to slides as described above. The fixed cells were permeabilized in excess fresh lysozyme solution (10 mg ml^-1^, 0.05 M EDTA, pH 8.0; 0.1 M Tris-HCl, pH 8.0) and the slides were incubated in a Thermo Brite^TM^ Slide Hybridization System (StatSpin, Abbott Molecular, Des Plaines, IL, USA) at 37°C for 1 h. The cells were washed by pipetting cold ultrapure water to the slide followed by absolute ethanol at a ∼20° angle and air-dried. Endogenous peroxidase was inactivated using 0.01 M HCl and slides were incubated for 10 min at room temperature (Sekar et al., 2003). The cells were dehydrated in 96% ethanol and air-dried. For the hybridization (35°C, 3 h), horseradish peroxidase labeled probe working stocks (50 ng μl^-1^; Biomers, Ulm, Germany) were diluted 1:100 in hybridization buffer (0.9 M NaCl, 20 mM Tris-HCl, 0.01% SDS, and 2% blocking reagent; Roche Diagnostic Boehringer, Basel, Switzerland) containing 55% formamide. The slides were washed (2 × 10 min) in pre-warmed washing buffer (13 mM NaCl, 20 mM Tris-HCl, 5 mM EDTA, and 0.01% SDS) in a Coplin jar placed in a 37°C water bath. After the washing, excess liquid was removed by dabbing the edge of the slide on Whatman paper and the slides were incubated in 1x PBS for 15 min at mild agitation and room temperature. Excess liquid was removed and 100 μl Cy3 TSA plus working solution (Perkin Elmer, Santa Clara, CA, USA) was pipetted onto the marked area of the slide. The slides were incubated for 3–10 min at room temperature in the dark. Slides were washed in 1x PBS and incubated again in 1x PBS for 15 min at mild agitation and room temperature followed by a rinse in cold ultrapure water and absolute ethanol as described above. The slides were air-dried, mounted with a drop of ProLong Diamond Antifade Mountant with DAPI (Molecular Probes, Eugene, OR, USA) and incubated at room temperature in the dark for 24 h.

Images were acquired on a Leica SP5 confocal microscope using a 63×/1.4 n.a. objective. The pixel size was set to 38.6 nm, oversampling Nyquist rates. A 405 nm laser was used to excite DAPI and light was collected between (415–485 nm). A 543 nm laser was used to excite Cy3 and light was collected between (555–625 nm). The scan rate was set between 10–100 Hz and eight line averaging was used. Gain was set for each channel to maximize dynamic range without saturating the signal. Cells were counted over an area of 32 mm^2^, corresponding to the size of the sort puddle, on tiled images acquired on a Zeiss AxioImager Z2 using a 63×/1.4 n.a. objective. Images were analyzed in Imaris ×64 version 8.1.2 as follows. DAPI or Cy3 stained objects were segmented using the Surfaces tool with background subtraction thresholds set to 10.5 and 6.28 for DAPI and Cy3 respectively. Segmented objects that were above 6.00 μm^2^ were included in the counts.

## Results

### Environmental Data

The water temperature during both summer and fall ranged between ∼14–17°C, which is characteristic of each season in Monterey Bay (Pennington and Chavez, 2000). Nutrient availability was generally high but there was variability among the sampling days (**Table 1**). Diatoms, including *Proboscia*, *Chaetoceros*, and *Leptocylindrus* dominated the phytoplankton community during the summer sampling (Supplementary Figure 2). In contrast, during the fall sampling, dinoflagellates dominated the phytoplankton community. The potentially toxic *Pseudo-nitzschia*, *Alexandrium*, and *Dinophysis* were observed during both summer and fall but were not dominant on our sampling dates. Flow cytometry showed that cell abundances of *Synechococcus* (see **Figure 1** and Supplementary Figure 1) were the lowest on July 9, with 1.1 × 10^3^ cells ml^-1^ and increased by one to two orders of magnitude in early October (**Table 1**). Compared to *Synechococcus*, the abundances of the photosynthetic picoeukaryote populations (P1 and P2; **Figure 1**) were less variable between the sampling dates and the abundances of the smaller population (P1) was generally one order of magnitude greater that the larger population (P2; **Table 1**).

**Table 1:** Environmental data for each sampling date and cell abundances as determined by flow cytometry for Syn (*Synechococcus*), P1 (photosynthetic picoeukaryotes with size ∼ <3 μm beads) and P2 (photosynthetic picoeukaryotes with size ∼ >3 μm beads).

Date	Temp (°C)	NO_3_ (μM)	PO_4_ (μM)	SiO_3_ (μM)	NH_4_ (μM)	Urea (μM)	Syn cells ml^-1^	P1 cells ml^-1^	P2 cells ml^-1^
June 18	14.9	1.04	0.24	4.31	0.89	0.31	na	na	na
July 2	14.1	0.05	0.06	1.64	0.82	0.20	na	na	na
July 9	15.6	1.78	0.89	23.07	5.37	0.63	1.1 × 10^3^	2.8 × 10^4^	3.2 × 10^3^
October 8	17.1	0.04	0.17	7.42	1.16	0.36	1.7 × 10^5^	2.3 × 10^4^	1.1 × 10^4^
October 15	16.0	2.68	0.95	9.11	5.58	2.14	5.9 × 10^4^	1.1 × 10^4^	1.6 × 10^3^
October 22	14.9	2.46	0.56	8.43	5.00	1.62	4.9 × 10^4^	1.3 × 10^4^	1.5 × 10^3^
October 29	15.3	1.93	1.42	14.39	4.04	0.85	4.2 × 10^4^	6.6 × 10^3^	6.1 × 10^2^

*na, data not available*.

### 16S rRNA Gene Clone Libraries

To investigate the presence of associated cells within the sorted photosynthetic picoeukaryote populations, 16S rRNA gene clone libraries were analyzed. For amplicons of the 27F and 1492R primer pair, only 2.7% of the sequences were non-Eukaryota (not mitochondrial or chloroplast). However, using the 895F and 1492R primer pair, designed to not amplify cyanobacteria and plastids, 31% of the sequences were non-Eukaryota (Supplementary Table 1). For the P2 sorts, only one non-Eukaryota sequence, a gammaproteobacterium 99% identical to an isolate from marine seabed sediments (accession number HE803906), was recovered. Thus, for subsequent analysis we focused on the P1 population.

**FIGURE 2:**
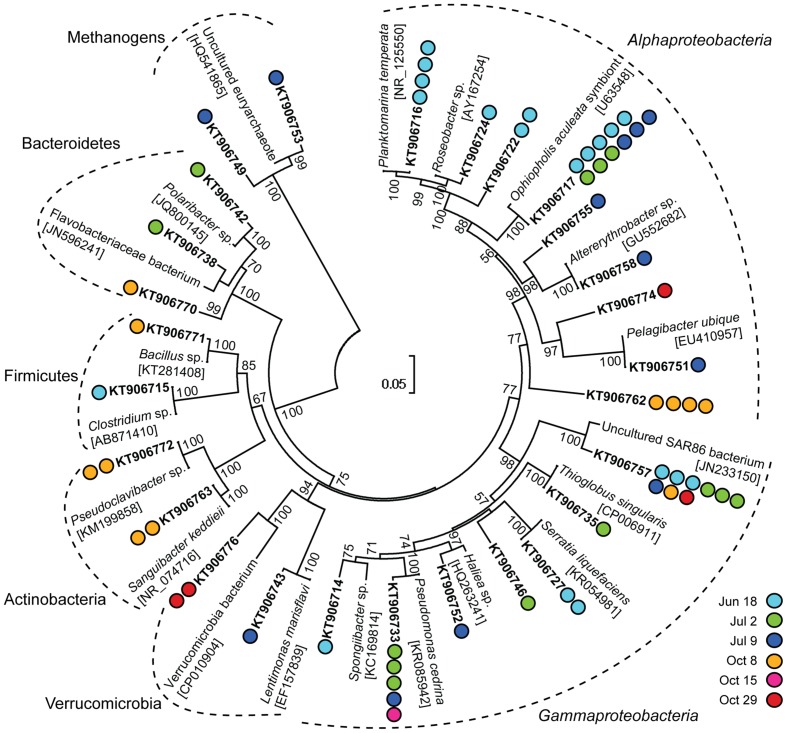
**Neighbor-joining phylogenetic tree of 97% similarity clustered non-Eukaryota sequences in 16S rRNA gene clone libraries from photosynthetic picoeukaryote cell sorts (P1, 1,000 cells) and their closest relatives**. GenBank accession numbers are written in square brackets for the closest relatives and in bold for the representative clones. Colored circles indicate the number of sequences in each cluster and the corresponding date for each clone. Bootstrap values >50% (1,000 replicates) are indicated next to the node.

The most frequently detected phylum among the non-Eukaryota sequences was Proteobacteria including *Alphaproteobacteria* and *Gammaproteobacteria* (**Figure 2**). Several clusters were detected on multiple sampling dates (**Figure 2**). In the summer samples, sequences within the *Roseobacter* clade were found. Members of the *Roseobacter* clade are one of the most abundant groups of pelagic bacterioplankton and have a central role in organic sulfur cycling (González et al., 1999). Within this clade, a cluster of sequences (KT906717; **Figure 2**) were 99% identical to a symbiont of the brittle star *Ophiopholis aculeata* (accession number U63548) and a Single Amplified Genome (SAG) from the Gulf of Maine (SAG ID MS024-1C; Stepanauskas and Sieracki, 2007). In the October 8 sample, an alphaproteobacterial cluster (KT906762; **Figure 2**) was 99% identical to a sequence from a photosynthetic picoeukaryote population sort from Station ALOHA in the Pacific Ocean (accession number EU187888; Zehr et al., 2008). The largest gammaproteobacterial cluster (KT906757; **Figure 2**) contained sequences from five sampling days and was related to SAR86, an abundant uncultivated marine bacterial clade that is metabolically streamlined (Dupont et al., 2012). In summary, the clone library results showed that sequences from associated bacterioplankton could be amplified from the sorted photosynthetic picoeukaryote populations.

### Isolates

In total, 16 bacterial isolates representing 12 phylotypes (>97% similarity clustering) were obtained from photosynthetic picoeukaryote cell sorts (P1) onto agar plates (**Table 2**). Three alphaproteobacterial isolates were closely related (99% identical) to *Sulfitobacter* sp., a member of the *Roseobacter* clade (**Table 2**). Four gammaproteobacterial isolates were 98% identical to members of the genus *Colwellia* and clustered with isolates from the starfish *Asterias amurensis* (accession number NR_116385; Choi et al., 2010) and a symbiont of deep-sea *Adipicola pacifica* mussels living attached to whale falls (accession number AB539012; Fujiwara et al., 2010). Isolates affiliated with typical opportunistic (r-strategist) bacteria such as *Alteromonas* and *Pseudomonas* were also obtained. From the sheath fluid puddles, seven isolates were obtained. These included three phylotypes that belonged to genera that have previously been reported as contaminants (e.g., Salter et al., 2014).

**Table 2:** List of isolates from this study and their affiliations.

Isolate number	Sort population	Number of sorted cells or volume	Closest cultivated relative	% Identity	Accession number	Classification
1	P1	1 cell	*Pseudomonas* sp.	98	AB180241	*Gammaproteobacteria*
2	P1	50 cells	*Colwellia asteriadis*	98	NR_116385	*Gammaproteobacteria*
3	P1	50 cells	*Staphylococcus pasteuri*	99	KP267845	*Firmicutes*
4	P1	50 cells	*Alteromonas stellipolaris*	99	AJ295715	*Gammaproteobacteria*
5	P1	50 cells	*Colwellia psychrerythraea*	98	CP000083	*Gammaproteobacteria*
6	P1	50 cells	*Arthrobacter* sp.	99	FR667186	*Actinobacteria*
7	P1	50 cells	*Lentimonas marisflavi*	98	EF157839	*Verrucomicrobia*
8	P1	100 cells	*Arthrobacter oxydans*	99	KP235208	*Actinobacteria*
9	P1	100 cells	*Colwellia asteriadis*	98	NR_116385	*Gammaproteobacteria*
10	P1	100 cells	*Sulfitobacter* sp.	99	KC160637	*Alphaproteobacteria*
11	P1	100 cells	*Altererythrobacter* sp.	97	JN848799	*Alphaproteobacteria*
12	P1	100 cells	*Sulfitobacter* sp.	99	KC160637	*Alphaproteobacteria*
13	P1	100 cells	*Sulfitobacter* sp.	99	KC428714	*Alphaproteobacteria*
14	P1	100 cells	*Novosphingobium* sp.	96	AY690709	*Alphaproteobacteria*
15	P1	1,000 cells	*Pseudoalteromonas citrea*	99	NR_037073	*Gammaproteobacteria*
16	P1	1,000 cells	*Colwellia* sp.	98	JF825446	*Gammaproteobacteria*
17	Sheath fluid	∼10 μl	*Ochrobactrum* sp.	99	KP410395	*Alphaproteobacteria*
18	Sheath fluid	∼10 μl	*Achromobacter* sp.	99	KT321695	*Betaproteobacteria*
19	Sheath fluid	∼10 μl	*Stenotrophomonas maltophilia*	99	GU254017	*Gammaproteobacteria*
20	Sheath fluid	∼10 μl	*Stenotrophomonas maltophilia*	99	GU254017	*Gammaproteobacteria*
21	Sheath fluid	∼10 μl	*Ochrobactrum* sp.	99	KP410395	*Alphaproteobacteria*
22	Sheath fluid	∼10 μl	*Achromobacter* sp.	99	KT321695	*Betaproteobacteria*
23	Sheath fluid	∼10 μl	*Achromobacter* sp.	99	KT321695	*Betaproteobacteria*

### High-Throughput 16S rRNA Gene Amplicon Sequence Libraries

**FIGURE 3:**
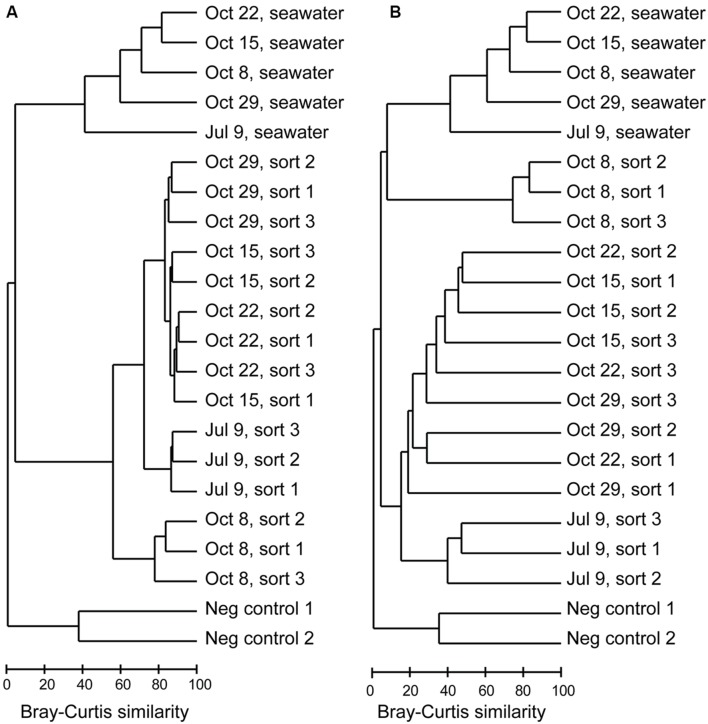
**Dendrograms based on Bray–Curtis similarity between Illumina MiSeq 16S rRNA gene libraries for **(A)** all OTUs, **(B)** non-Eukaryota OTUs**.

**FIGURE 4:**
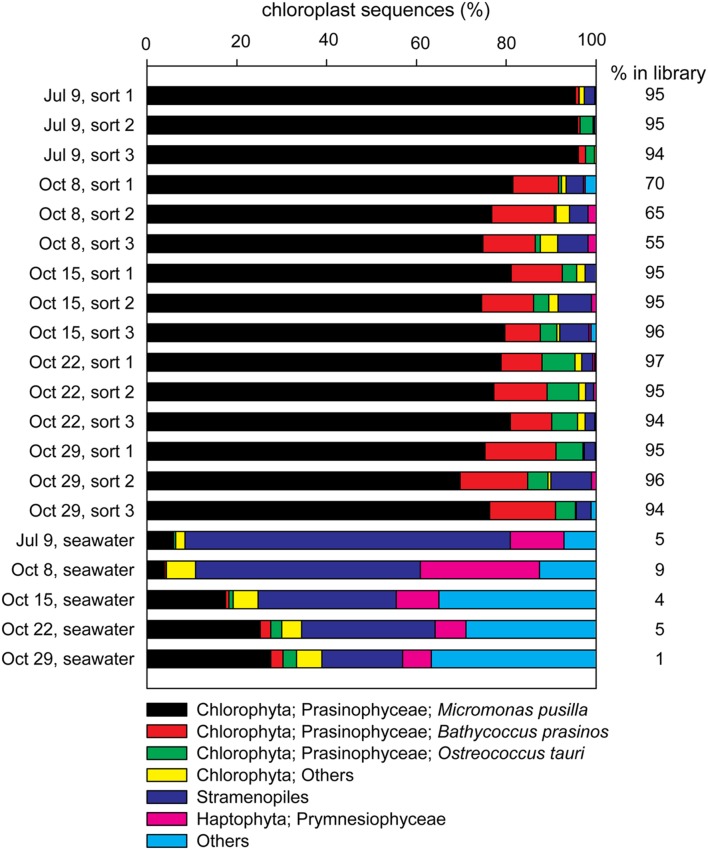
**Contributions of the most frequently occurring eukaryotic phyla among chloroplast sequences detected in each of the sorted photosynthetic picoeukaryote samples (1,000 P1 cells, triplicate samples) and the corresponding seawater samples (0.2–10 μm) for each sampling date**. The percentage of chloroplast sequences within each library is indicated to the right of each bar.

16S rRNA gene amplicons from triplicate samples of 1,000 photosynthetic picoeukaryote cell sorts (P1) and the corresponding bulk seawater samples from five sampling occasions were sequenced (**Table 3**). The composition of OTUs among the triplicate sorts were more similar to each other compared to samples from a different day but the adjacent sampling dates October 15, October 22, and October 29 showed high Bray–Curtis similarity (>80%; **Figure 3A**). Most of the sequences in the photosynthetic picoeukaryote sort samples were plastid (**Figure 4**). A large cluster of OTUs were 96–100% identical to *Micromonas pusilla* and these sequences were most frequently detected in all sorted samples (accounting for between 75–96% of chloroplast sequences). *Micromonas*, a marine prasinophyte within the order Mamiellales, are known to be diverse and globally distributed (Guillou et al., 2004; Worden et al., 2009). *Bathycoccus prasinos* and *Ostreococcus tauri* OTUs were also detected in all samples. Notably, at lower relative abundances in the summer sample compared to the fall samples (**Figure 4**). In comparison to the sorted samples, the chloroplast sequences from bulk seawater were more diverse and not solely dominated by Chlorophyta. For example, in the bulk seawater samples, Haptophytes and Stramenopiles were present at high relative abundances (18–24%) while these phyla composed <10% in all of the sorted samples. The photosynthetic picoeukaryote populations were sorted based on chlorophyll *a* content and size. Thereby larger eukaryotes (∼ >3 μm beads) as well as heterotrophic picoeukaryotes would be excluded unless they had attached or ingested chlorophyll *a* containing cells. Thus, a difference in composition and a greater diversity of eukaryotic cells was expected in the bulk seawater samples compared to the sorted samples (**Figures 3A** and **4**).

**Table 3:** Total number of sequences and OTUs (97% similarity) with >10 sequences obtained using Illumina MiSeq for each sample.

Date	Sample	Number of sequences	Number of OTUs	Number of non-Eukaryota sequences	Number of non-Eukaryota OTUs
July 9	P1, 1,000 cells	22,871	501	1,055	60
July 9	P1, 1,000 cells	36,107	558	1,577	67
July 9	P1, 1,000 cells	39,811	621	1,990	88
October 8	P1, 1,000 cells	60,253	735	17,485	171
October 8	P1, 1,000 cells	44,326	637	14,718	148
October 8	P1, 1,000 cells	49,950	676	21,439	195
October 15	P1, 1,000 cells	48,500	649	1,219	70
October 15	P1, 1,000 cells	46,421	671	1,279	82
October 15	P1, 1,000 cells	50,868	665	1,226	71
October 22	P1, 1,000 cells	51,827	644	618	44
October 22	P1, 1,000 cells	66,302	729	1,447	79
October 22	P1, 1,000 cells	81,184	761	2,202	103
October 29	P1, 1,000 cells	38,845	570	780	36
October 29	P1, 1,000 cells	54,227	647	773	37
October 29	P1, 1,000 cells	26,428	478	1,137	44
July 9	Seawater	70,141	923	63,683	812
October 8	Seawater	69,548	1,172	60,703	1,036
October 15	Seawater	56,095	1,156	51,657	1,035
October 22	Seawater	73,012	1,250	65,462	1,063
October 29	Seawater	63,590	1,120	60,092	1,041
–	Negative PCR control 1	42	5	9	4
–	Negative PCR control 2	49	14	25	12
**Total**		1,050,488	2,352	370,576	1,372

**FIGURE 5:**
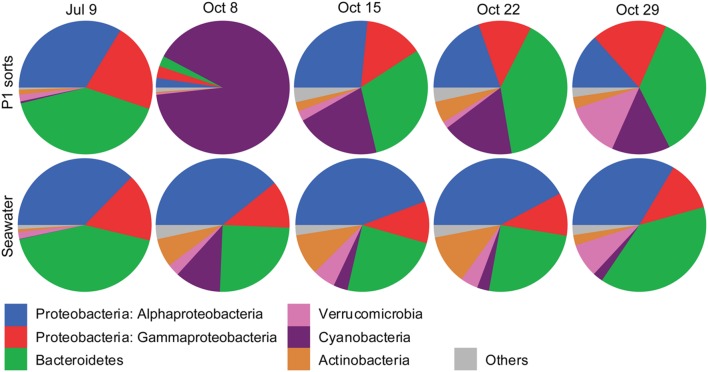
**Pie graphs showing the phylogenetic affiliations of non-Eukaryota 16S rRNA gene sequences in photosynthetic picoeukaryote sorts (P1, 1,000 cells), and bulk seawater (0.2–10 μm) from Illumina MiSeq libraries for each sampling date**. The absolute numbers of sequences for the triplicate sort samples from each sampling date have been pooled. The colors for each phyla are indicated below the graphs.

**FIGURE 6:**
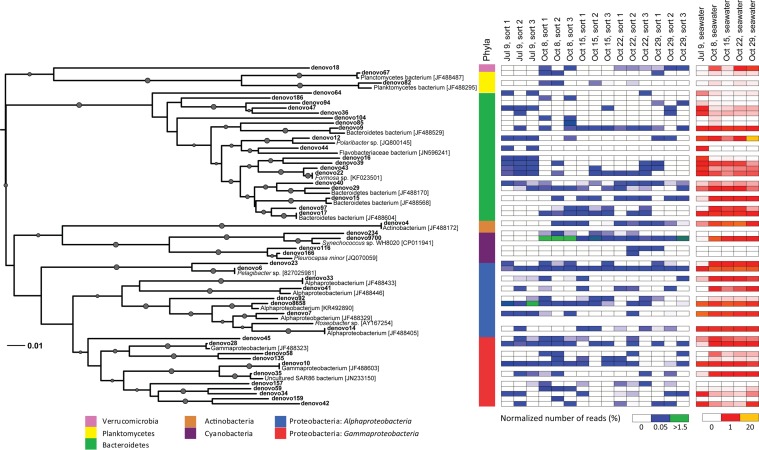
**Neighbor-joining phylogenetic tree of non-Eukaryota OTUs (97% similarity) in 16S rRNA gene Illumina MiSeq libraries with >100 sequences present in photosynthetic picoeukaryote cell sorts (P1, 1,000 cells)**. OTUs are indicated in bold and GenBank accession numbers for the closest relatives are written in square brackets. Bootstrap values >50% (1,000 replicates) are indicated by circles with the size of the circles corresponding to the bootstrap value. A colored bar shows which phyla the branches of the tree are affiliated with. Heatmaps for photosynthetic picoeukaryote sort (P1, 1,000 cells) and seawater samples show the normalized number of sequences of each OTU in the different samples. The *Synechococcus* clade (18 OTUs, >96% similarity) has been collapsed for clarity and is represented by denovo9700.

The Illumina MiSeq sequencing confirmed the presence of non-Eukaryota cells among the sorted photosynthetic picoeukaryote cells. In total, 1,372 OTUs of non-Eukaryota origin were present in the dataset ranging from between 36 and 103 OTUs present in each sorted sample (**Table 3**). The most frequently detected phyla in the photosynthetic picoeukaryote sorts were Proteobacteria (*Alphaproteobacteria* and *Gammaproteobacteria*), Bacteroidetes, and Cyanobacteria, corresponding to the main phyla in the bulk seawater samples (**Figure 5**) but the OTU composition was different in the sorted samples compared to the bulk seawater samples (**Figure 3B**). In general, there was patchiness in the presence/abundance of non-Eukaryota OTUs between replicates even among the OTUs with highest relative abundances (**Figures 3B** and **6**). The patchiness between the biological replicates indicate that there is a large variation in the associated microbes and that larger population sorts will be required in order to reliably quantify the occurrences of specific taxa.

Among the cyanobacterial OTUs in photosynthetic picoeukaryote sorts, *Synechococcus*-like OTUs were most frequently detected (**Figure 6**). The *Synechococcus* OTU with the highest relative abundance was 98% identical to *Synechococcus* sp. WH 8020; a member of sub-cluster 5.1B clade I which has been described as a coastal and opportunist strain (Dufresne et al., 2008). In the October 8 sample, when the highest observed abundance of *Synechococcus* occurred in the bulk water samples (**Table 1**), there was a dominance of *Synechococcus* sequences in the sorted photosynthetic picoeukaryote populations (90.5% ± 0.3 of sequences; **Figure 5**). Thereby, they were more similar in OTU composition to the bulk seawater samples compared to the other sorted samples (**Figure 3B**). The separation of *Synechococcus* from the photosynthetic picoeukaryote population was done based on orange fluorescence. Depending on the definition of the sorting gates, some *Synechococcus* may have been co-sorted if the gate for the orange fluorescence was defined too stringently. However, if gates would be defined more broadly (i.e., excluding all orange fluorescence cells) other phycoerythrin containing picoeukaryotic cells such as some Cryptophytes would also be excluded. Since the relative abundances of *Synechococcus* in the October 8 sorts were very high in comparison to other sampling dates, it cannot be dismissed that this may have resulted from not fully excluding the *Synechococcus* population from the photosynthetic picoeukaryote sorts using the NOT gate. The inclusion of these cells could potentially influence the composition of non-Eukaryota sequences in the October 8 sorts. However, because of the stringency used for the flow cytometry sorting we consider co-sorting events from other populations to be unlikely.

The *Pelagibacter*-like OTU (denovo6) was consistently present in all samples and was the second most frequently detected alphaproteobacterial OTU (**Figure 6**). The most frequently detected alphaproteobacterial OTU (denovo8658) was 98% identical to a symbiont of the brittle star *Ophiopholis aculeata* (accession number U63548), the same closest relative as for clone KT906717 which was detected in all summer samples (**Figure 2**). Notably, all isolates from photosynthetic picoeukaryote sorts in this study had closely related OTUs (94–99% identity) in the Illumina MiSeq dataset (Supplementary Table 2). For example, nine OTUs clustered within the *Roseobacter* clade and an OTU present in a sort from July 9 (denovo87, 34 sequences) was 96–98% identical to the *Sulfitobacter* sp. isolates (Supplementary Table 2) suggesting that this may be another interesting alphaproteobacterial target. Several gammaproteobacterial OTUs (e.g., denovo59) were consistently present in the triplicate sort samples from specific sampling dates even though the relative abundances in bulk seawater were low (**Figure 6**), making them interesting targets for future studies. The most frequently detected gammaproteobacterial OTU in the sorted samples (denovo10) was 100% identical to a group of 12 SAGs isolated from the Gulf of Maine (accession number JF488603; Martinez-Garcia et al., 2012b). In contrast to the clone libraries, OTUs within the SAR86-like cluster were present but showed no indication of being enriched in the sorted populations as they were not detected on several occasions, even when the relative abundance in bulk seawater was high (>1%; **Figure 6**).

The Bacteroidetes sequences were diverse and several OTUs were consistently present in July 9 sorts (**Figure 6**). The Bacteroidetes OTUs were dominated by *Flavobacteria* with the two most prominent OTUs (denovo9 and denovo15) 99% identical to SAGs isolated from the Gulf of Maine (accession numbers JF488529 and JF488568; Martinez-Garcia et al., 2012b). The only actinobacterial OTU with >100 sequences (denovo4) was 100% identical to a SAG from the same study (accession number JF488172; Martinez-Garcia et al., 2012b) and was detected in all of the October samples (**Figure 6**). Interestingly, the closest relatives of many of the other dominant OTUs also had high similarity to SAGs reported by Martinez-Garcia et al. (2012b; see closest relatives with accession numbers starting with JF488 in **Figure 6**).

Molecular analyses of samples with low DNA concentrations require extra precautions to rule out contamination. To investigate the possibility of reagent contamination two negative PCR controls were sequenced together with the samples on the Illumina MiSeq platform. The recovery of sequences from the negative PCR controls was poor (<100 sequences total, **Table 3**) and the overlap with the environmental samples was minimal (six OTUs; **Figure 3**). One sequence from the negative PCR control clustered with the most abundant *Synechococcus* sp. OTU (49,249 sequences total), which was likely the result of a technical mis-assignment associated with Illumina MiSeq sequencing. The other overlapping OTUs consisted of Proteobacteria and Actinobacteria and represented <0.05% of the total sequences. These OTUs were excluded from the taxonomic and phylogenetic analyses (**Figures 5** and **6**). Within the samples, eight clusters (corresponding to 0.1% of total sequences) were classified with sequences that were not similar to sequences found in environmental studies (e.g., *Mycoplasma* and *Staphylococcus*). The finding of such non-environmental sequences is not uncommon in studies using similar approaches (e.g., Baker and Kemp, 2014). Interestingly, the majority (94%) of these sequences were from one sample (October 22, sort 3) indicating that this was not a systematic problem. Notably, the isolates from sheath fluid did not cluster (<97% similarity) with any of the sequences in the Illumina MiSeq dataset suggesting that the sheath fluid and fluidic system were not a source of contamination in the sorted samples. Based on the limited overlap and few occurrences of negative control sequences, we conclude that putative contamination in our study was limited and the finding of diverse bacteria in association with photosynthetic picoeukaryote populations was not due to contamination.

### Microscopy

For the CARD-FISH protocol, initial tests and protocol optimization was done using four isolates from this study. The EUB338 probe labeled cells from all cultures while the GAM42a probe only labeled the gammaproteobacterial cultures. The no probe and the non338 probe controls indicated that autofluorescence signal was low compared to the Cy3 signal and could easily be distinguished. The EUB338 probe labeled 43% of the sorted photosynthetic picoeukaryote cells (P1, 1,770 cells were counted in the Cy3 channel and 4,156 cells in the DAPI channel). The average cell diameter of the cells with both DAPI and Cy3 signal was 3.9 μm with a standard deviation of 2.6 μm and the median value of the cell diameter was 2.8 μm. Morphological cell differences were observed with a dominance of spherical, followed by crescent shaped cells. Since the EUB338 probe is known to hybridize with chloroplasts of several marine picoeukaryotes, e.g., *Micromonas*, *Bathycoccus*, and *Ostreococcus* (NCBI Blastn), it is likely that a large fraction of the observations were eukaryotic organelles (Supplementary Figure 3). In an attempt to visualize the associated bacteria, the GAM42a probe, a probe that is more specific and less likely to hybridize to picoeukaryotes (Biegala et al., 2005), was used. In total three slides were screened but no hybridization with the GAM42a probe was observed within the sorted photosynthetic picoeukaryote populations.

## Discussion

Associations between bacterioplankton and picoeukaryotes are thought to frequently occur in the marine environment, but are thus far poorly characterized. In this study, a combination of flow cytometry sorting and sequencing, as well as bacterial isolation on agar plates, was used to demonstrate that diverse bacterioplankton were present within photosynthetic picoeukaryote populations. Previous studies showed that the association between the nitrogen-fixing unicellular cyanobacterium UCYN-A and its prymnesiophyte host were easily dislodged if seawater samples were concentrated (Thompson et al., 2012). Therefore all sorts in this study were done using fresh, unpreserved seawater samples. In this study, extensive efforts were taken to minimize and identify methodological biases as well as putative contamination. The sequencing of negative PCR controls showed no indications of an external source of the detected non-Eukaryota sequences. Considering that the sorting was done in the 1.0 drop purity-mode, based on red fluorescence, a non-chlorophyll *a* cell can theoretically only be co-sorted if it is attached to or within a chlorophyll *a* containing cell. However, despite the strict sorting criteria used in this study, the complete absence of co-sorting of unattached bacteria cannot be certain.

As expected from a coastal site with high nitrate concentrations, the abundances of photosynthetic picoeukaryotes (P1) were high on all sampling occasions (**Table 1**). The most frequently occurring chloroplast sequences were affiliated with *Micromonas pusilla* but *Bathycoccus* and *Ostreococcus* were also detected (**Figure 4**), corresponding to the most significant picoeukaryotes in coastal areas (Massana, 2011). Interestingly, the bacterial OTUs with the highest relative abundances in the bulk seawater samples were also present in the photosynthetic picoeukaryote sort samples (**Figure 6**). Although we cannot rule out the possibility of these being a result of co-sorting, an intriguing possibility is that these dominating taxa in the free-living community could have been ingested through bacterivory. An increasing number of environmental studies suggest that mixotrophy may be widespread among photosynthetic picoeukaryotes (Zubkov and Tarran, 2008; Sanders and Gast, 2012; Hartmann et al., 2013). In a recent study, using a dual CARD-FISH protocol, Prymnesiophyceae, Chrysophyceae, and Pelagophyceae cells from the Atlantic Ocean showed internalization with *Prochlorococcus* and SAR11 cells (Hartmann et al., 2013). Hartmann et al. (2013) concluded that because *Prochlorococcus* or SAR11 are considered free-living genera, the presence of these cells inside the eukaryote cells indicated bacterivory. Bacterivory has also been demonstrated for a *Micromonas pusilla* culture (González et al., 1993) and a recent study suggests that *Micromonas* was selective for small size pray, represented by 0.5 μm microspheres, while larger microspheres (0.9 μm) were also ingested, albeit at a lower rate (McKie-Krisberg and Sanders, 2014). Thus, if bacterivory was widespread within these natural populations of photosynthetic picoeukaryotes it can be expected that grazing strategy and selectivity will affect the non-Eukaryota sequences present within the photosynthetic picoeukaryote sorts (Worden et al., 2015).

The composition of non-Eukaryota OTUs in the sorted samples was different from the bulk seawater samples (**Figure 3B**). With an exception of the October 8 sorts, which consisted of a dominating *Synechococcus* OTU (**Figure 5**, <20% Bray–Curtis similarity **Figure 3B**), the sorted samples had <10% Bray–Curtis similarity compared with the bulk seawater samples (**Figure 3B**) suggesting that specific phylotypes were enriched in the sorted populations and thereby may represent functional associations between bacteria and photosynthetic picoeukaryotes. Although the probability is low, the sequencing of several samples simultaneously may result in a mis-assignment of reads to the wrong sample (e.g., Kircher et al., 2012; Mitra et al., 2015). By limiting our phylogenetic analysis to OTUs with >100 sequences within the photosynthetic picoeukaryote sorts (**Figure 6**), we view such occurrences as unlikely to influence the findings of this study.

Similarly to what has been observed in a wide range of diatom–bacterial interactions (Amin et al., 2012), the most frequently detected bacterioplankton phyla within the sorted photosynthetic picoeukaryote populations were Proteobacteria (*Alpha*- and *Gammaproteobacteria*) and Bacteroidetes (**Figures 2** and **5**). *Gammaproteobacteria* and Bacteroidetes also predominated bacterial 16S rRNA gene sequences recovered from mixotrophic protist SAGs and *Gammaproteobacteria* were found to be more likely to be association with protists in comparison to the free bacterioplankton SAGs (Martinez-Garcia et al., 2012a). Although not directly comparable because of the different sequencing regions, the clone libraries in this study also suggest that Proteobacteria may be prevalent in association with photosynthetic picoeukaryotes. An interesting alphaproteobacterial cluster of clone sequences (KT906717) was observed in all summer samples (**Figure 2**) and the second most frequently detected alphaproteobacterial OTU (denovo8658; **Figure 6**) were both 98% identical to a bacterial symbiont of the brittle star *Ophiopholis aculeata* (accession number U63548). The re-occurrence of this phylotype and its high relative abundances in comparison to other non-Eukaryota OTUs within the sorted photosynthetic picoeukaryote populations suggests that it may be an interesting target for further characterization.

The composition of the non-Eukaryota phylotypes detected in the photosynthetic picoeukaryote populations was likely influenced by the design of this study. For example, by the definition of the sort gate, cells associated with larger photosynthetic picoeukaryotes were excluded since only the smaller size-fraction (∼ <3 μm beads) was examined. In addition, NGS sequencing of amplicons is associated with regular PCR biases which influence the observed community composition. In the Illumina MiSeq libraries, triplicate cell sorts from the same seawater samples were sequenced and the lack of replication between the samples was surprising (**Figure 6**). This is an indication that associated non-Eukaryota cells are likely of low abundance within the photosynthetic picoeukaryote populations and may be highly diverse. However, the re-occurrence of specific OTUs on different sampling days suggests that the associations with these phylotypes were prevalent. The different patterns of non-Eukaryota OTUs observed between bulk seawater and sorted photosynthetic picoeukaryote populations indicate that co-sorting plays a negligible role in this study. Furthermore, many of the OTUs detected in this study, distributed over different phyla, were most identical to sequences reported from SAGs from the Gulf of Maine (see accession numbers starting with JF488 in **Figure 6**; Martinez-Garcia et al., 2012a) rather than sequences reported from seawater studies which are numerically dominating in databases. This is an indication that flow-cytometry sorting and downstream molecular analysis more easily accesses parts of the microbial community which are not generally captured when bulk seawater samples are observed.

Some aquatic bacteria have complex lifestyles and may quickly alternate between free-living and associated stages, exploiting nutrient rich microenvironments in association with phytoplankton (Grossart, 2010). For example, dead phytoplankton cells may be quickly colonized and enzymatically degraded by bacteria (Azam and Malfatti, 2007). The high nutrient cultivation plates used in this study likely selected for bacteria that thrive under nutrient rich conditions. Interestingly, all of the isolates had closely related, but generally not numerically dominant, OTUs within the Illumina MiSeq libraries (Supplementary Table 2). Thus indicating that bacteria present in association with photosynthetic picoeukaryotes were isolated using this method. Several of the isolates obtained in this study were close relatives to bacteria that are known to thrive on algal surfaces and/or on aggregates. An OTU 97% identical to the *Alteromonas* sp. isolate was observed in two photosynthetic picoeukaryote sorts (Supplementary Table 2). *Alteromonas* sp. is an *r*-strategist *Gammaproteobacteria* that is known to quickly exploit nutrient rich hot spots in the environment (Fontanez et al., 2015) and have frequently been reported in association with protists (Su et al., 2011). Four isolates in this study were closely related to *Colwellia* (**Table 2**). Although *Colwellia psychrerythraea* has been described as a facultative anaerobic psychrophile (Methé et al., 2005), a sequence similar to *Colwellia psychrerythraea* was detected in aggregates from the Santa Barbara Channel (DeLong et al., 1993), suggesting that there could be an adaptation for an associated lifestyle within *Colwellia*. In the Pacific Ocean, *Sulfitobacter* sp., a member of the alphaproteobacterial *Roseobacter* clade, was a rapid colonizer of sinking marine aggregates (LeCleir et al., 2014). In this study, three *Sulfitobacter* sp. isolates were obtained from 100 photosynthetic picoeukaryote cells (**Table 2**) and an OTU with 96–98% identity was detected in all seawater samples and in one sort sample (Supplementary Table 2). *Sulfitobacter* are known for diverse species-specific associations with marine eukaryotes (Jasti et al., 2005; Amin et al., 2012). Recently, four *Sulfitobacter* strains were shown to promote cell division in the coastal diatom *Pseudo*-*nitzschia multiseries* (Amin et al., 2015). The authors used transcriptomics and targeted metabolite analysis to characterize the association and suggested that a complex exchange of nutrients took place between the diatom–bacteria consortia. It is possible that the isolates obtained in this study were involved in similar interactions but further studies are needed to assess if these associations are purely opportunistic or if there was species-specific adaptation.

In addition to being ingested or surface attached to the phytoplankton cell, associated cells may be present intracellularly as endosymbionts. Endosymbionts have been described within the cultured heterotrophic picoeukaryote *Symbiomonas scintillans* (Guillou et al., 1999) but to what extent endosymbionts may be present within photosynthetic picoeukaryotes is not well known. To try to visualize bacteria associated with photosynthetic picoeukaryotes we developed an on-slide CARD-FISH protocol on flow cytometry sorted photosynthetic picoeukaryote cells. We used the EUB338 probe that has been widely used for enumerating bacteria in seawater and in association with algae (e.g., Tujula et al., 2006). However, similarly to the observations by Biegala et al. (2005), the hybridization of the EUB338 probe to a large fraction of the picoeukaryote cells caused difficulties in distinguishing associated cells from chloroplasts (Supplementary Figure 3). Biegala et al. (2005) found hybridization associated with picoeukaryotes using the more specific probe targeting *Gammaproteobacteria* (GAM42a), which is unlikely to hybridize to eukaryotic organelles. However, when using the GAM42a probe in this study, no hybridized cells were observed within the photosynthetic picoeukaryote sorts. Although the developed on-slide CARD-FISH protocol was successful in labeling cells from sorts, providing information about the cell size and morphology of the sorted population, it was a low-throughput method because of the low number of cells visible on the slides (<30% of the sorted cells). Difficulties associated with sorting raw seawater such as low abundances and the small cell size of the sorted populations also made identification and replication challenging. In the future, optimization of the protocol to prevent cell loss and the use of several more specific probes targeting for example *Synechococcus* will likely enable visualization of associated microbes.

In contrast to associations by opportunistic colonizers on dead or alive phytoplankton cells, some symbioses may have co-evolved for a long period of time and exhibit extreme genome reductions with metabolic pathways missing in one partner but present in the other (McCutcheon and Moran, 2012). However, to establish the metabolic processes and benefits gained by the associated organisms can be challenging, especially if the associated organism have not been cultured. In a recent study, the microbiome of non-axenic *Ostreococcus tauri* cultures was studied and specific genes involved in cell-to cell-interactions identified (Abby et al., 2014). In the future, similar studies may expand the microbial tool kit of putative targets that are much needed to study bacterioplankton–picoeukaryote associations *in situ*. In the light of recent discoveries, it is not unlikely that ecological associations between bacterioplankton and picoeukaryotes are diverse and frequently occurring. Consequently, identifying, characterizing and understanding the metabolic mechanisms underlying these interactions is a prerequisite in order to improve community modeling approaches. Here, we identify specific phylotypes found in association with photosynthetic picoeukaryotes which can be used as targets for further characterization and visualization of the associations.

## Author Contributions

HF, KT-K, and JZ designed the experiments. HF performed the experiments. HF, KT-K, and JZ wrote the paper.

## Conflict of Interest Statement

The authors declare that the research was conducted in the absence of any commercial or financial relationships that could be construed as a potential conflict of interest.

## Footnotes

1 http://mcb.berkeley.edu/barker/dnaseq/

2 www.decipher.cee.wisc.edu
