# Supplementary material for: Identification of Associations between Bacterioplankton and Photosynthetic Picoeukaryotes in Coastal Waters
Source: Front Microbiol. 2016 Mar 22;7:339. doi: 10.3389/fmicb.2016.00339 (PMC4834442; doi:10.3389/fmicb.2016.00339)
Supplement: Supplementary file 1 [file Supplementary_Material.DOCX]

Supplementary Material

**Identification of Associations between Bacterioplankton and Photosynthetic Picoeukaryotes in Coastal Waters**

**Hanna Farnelid^*^, Kendra Turk-Kubo, Jonathan Zehr**

***Correspondence:** Hanna Farnelid: hannafarnelid@gmail.com

# Supplementary Figures and Tables

## Supplementary Figures

**
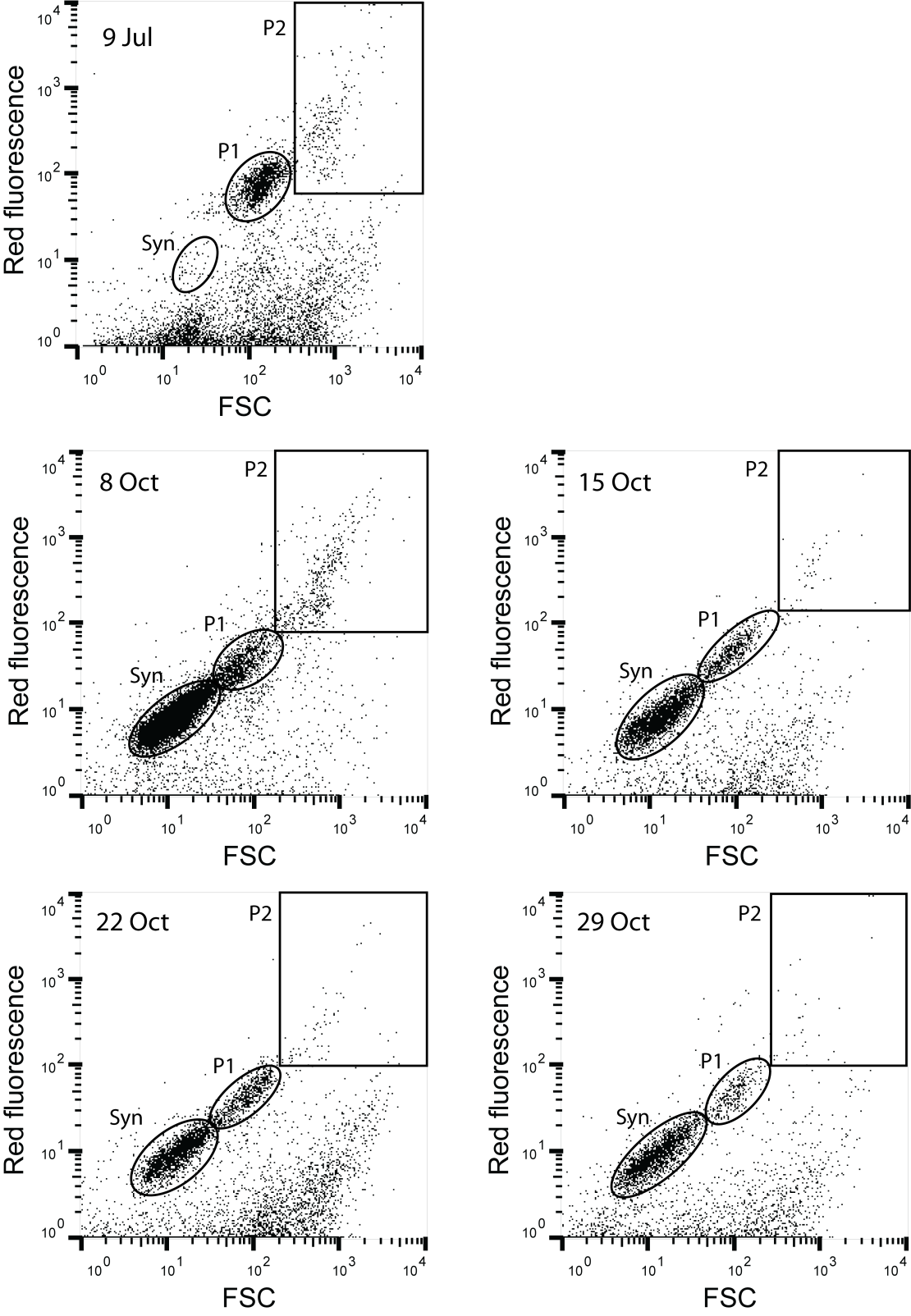
**

**Supplementary Figure 1.** Flow cytograms showing 100 000 events and the targeted cell populations on all sampling dates except for Jun 18 and Jul 2. Red fluorescence is a proxy for chlorophyll a content and forward scatter (FSC) is a proxy for cell size. All samples are unpreserved and not concentrated. The indicated cell populations are *Synechococcus* (Syn), smaller photosynthetic picoeukaryotes (P1) and larger photosynthetic picoeukaryotes (P2). *Synechococcus* were defined and distinguished from the P1 populations based on orange fluorescence as a proxy for phycoerythrin content. Note that the *Synechococcus* population was almost absent on Jul 9 while it was a distinct and dominant group during the fall sampling.


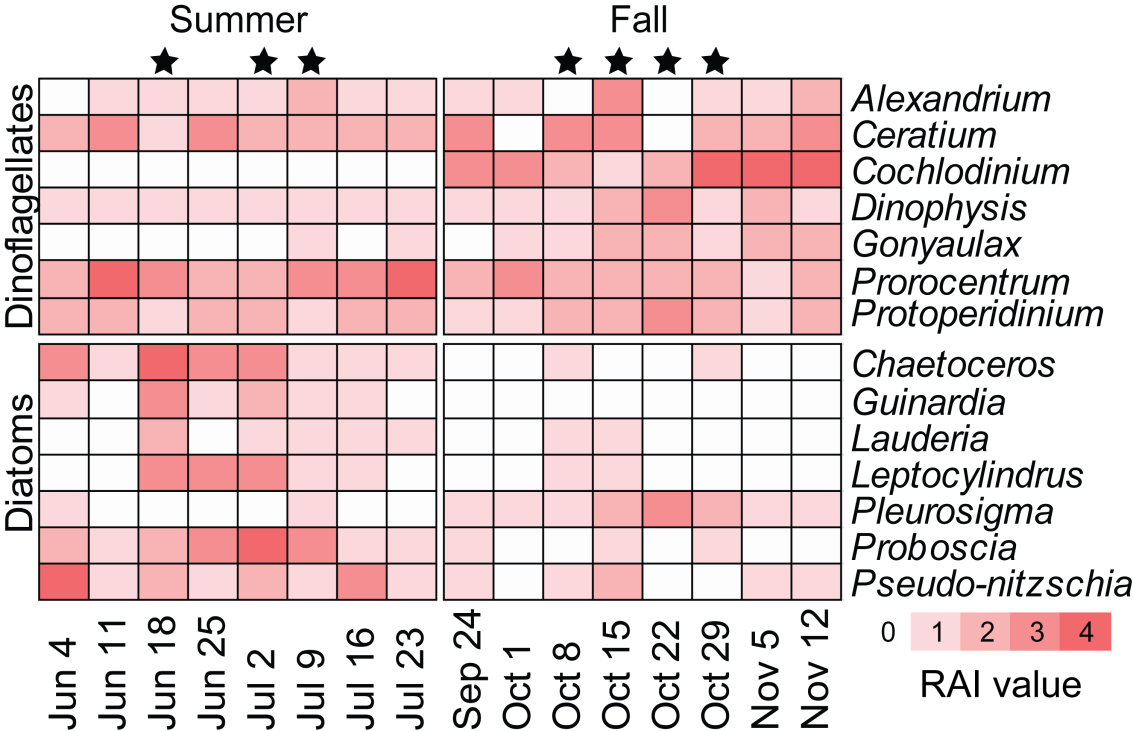


**Supplementary Figure 2:** Relative Abundance Indexes (RAI) of the most frequently observed diatom and dinoflagellate genera during the periods of sampling in summer and fall 2014. The RAI varies from not observed to dominant and is shown by a heatmap with increasing red color.

**
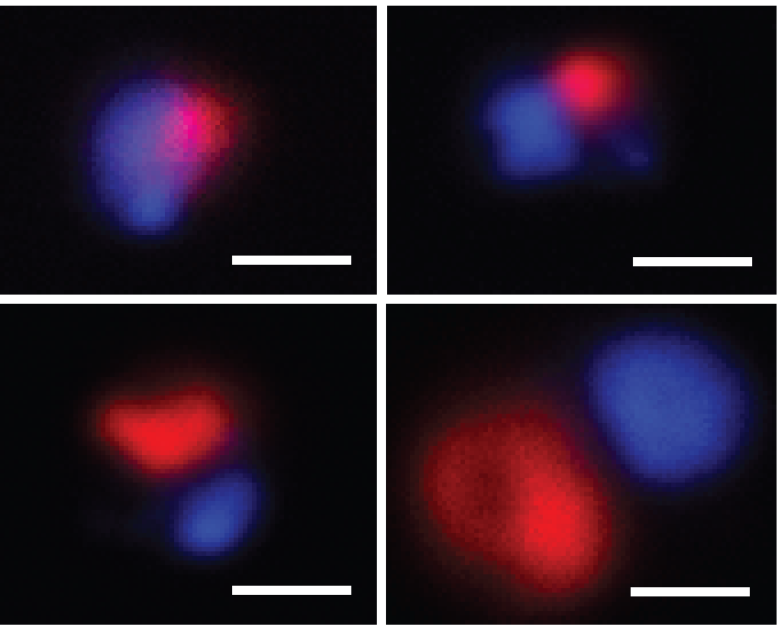
**

**Supplementary Figure 3.** Confocal micrographs of sorted photosynthetic picoeukaryote cells. Images are overlays between hybridized (EUB338 probe; red) and DAPI-stained cells (blue). Scale bar = 1 µm.

## Supplementary Tables

**Supplementary Table 1:** Summary of cloned amplicons from sorted populations, primer sets used and number of sequences. The sorted populations were P1 and P2 (photosynthetic picoeukaryotes), and Syn (*Synechoccocus*).

| Date | Population | Number of sorted cells | Primer set | Number of sequences | | | | |
| --- | --- | --- | --- | --- | --- | --- | --- | --- |
|  |  |  |  | Non-Eukaryota | Plastid | Mitochondrial | Undefined Eukaryotic | Total |
| 18-Jun | P1 | 1000 | 895F/1492R | 19 | 2 | 1 | 0 | 22 |
| 18-Jun | P2 | 250 | 895F/1492R | 1 | 2 | 21 | 0 | 24 |
| 02-Jul | P1 | 1000 | 895F/1492R | 14 | 6 | 3 | 0 | 23 |
| 02-Jul | P2 | 100 | 895F/1492R | 0 | 2 | 3 | 0 | 5 |
| 09-Jul | P1 | 1000 | 895F/1492R | 9 | 11 | 0 | 1 | 21 |
| 09-Jul | P1 | 1000 | 27F/1492R | 3 | 73 | 0 | 0 | 76 |
| 09-Jul | P2 | 100 | 895F/1492R | 0 | 2 | 3 | 0 | 5 |
| 09-Jul | Syn | 100 | 27F/1492R | 3 | 0 | 0 | 0 | 3 |
| 08-Oct | P1 | 1000 | 895F/1492R | 11 | 10 | 0 | 0 | 21 |
| 15-Oct | P1 | 1000 | 895F/1492R | 1 | 17 | 4 | 0 | 22 |
| 22-Oct | P1 | 1000 | 895F/1492R | 0 | 21 | 3 | 0 | 24 |
| 29-Oct | P1 | 1000 | 895F/1492R | 4 | 12 | 1 | 2 | 19 |
| Total number of sequences | | |  | 65 | 158 | 39 | 3 | 265 |

**Supplementary Table 2.** Isolates and their closest OTU within the 16S rRNA gene Illumina MiSeq libraries and the number of reads for each sample.

| Isolate number | Closest OTU | Identity | Closest relative of isolate | Classification | Jul 9  sorts | | | Oct 8  sorts | | | Oct 15  sorts | | | Oct 22  Sorts | | | Oct 29  sorts | | | Jul 9  seawater | Oct 8  seawater | Oct 15  seawater | Oct 22  seawater | Oct 29  seawater |
| --- | --- | --- | --- | --- | --- | --- | --- | --- | --- | --- | --- | --- | --- | --- | --- | --- | --- | --- | --- | --- | --- | --- | --- | --- |
| 10, 12, 13 | denovo 87 | 96-98% | *Sulfitobacter* sp. | *Alphaproteobacteria* | 0 | 0 | 34 | 0 | 0 | 0 | 0 | 0 | 0 | 0 | 0 | 0 | 0 | 0 | 0 | 206 | 21 | 77 | 175 | 69 |
| 11 | denovo749 | 96% | *Alterethrobacter* sp. | *Alphaproteobacteria* | 0 | 0 | 0 | 0 | 0 | 6 | 0 | 0 | 0 | 0 | 0 | 0 | 0 | 0 | 0 | 0 | 40 | 1 | 5 | 3 |
| 14 | denovo7927 | 99% | *Novosphingobium sp.* | *Alphaproteobacteria* | 0 | 2 | 0 | 0 | 0 | 1 | 1 | 0 | 0 | 0 | 0 | 0 | 0 | 0 | 0 | 0 | 5 | 0 | 3 | 1 |
| 2, 5, 9, 16 | denovo11706 | 95-97% | *Colwellia asteriadis* | *Gammaproteobacteria* | 0 | 0 | 0 | 0 | 0 | 0 | 0 | 0 | 0 | 0 | 0 | 0 | 0 | 0 | 0 | 0 | 0 | 0 | 1 | 1 |
| 15 | denovo560 | 98% | *Pseudoalteromonas* | *Gammaproteobacteria* | 0 | 0 | 0 | 0 | 0 | 0 | 0 | 0 | 0 | 0 | 0 | 0 | 0 | 0 | 0 | 3 | 2 | 4 | 12 | 6 |
| 4 | denovo79 | 97% | *Alteromonas* | *Gammaproteobacteria* | 35 | 0 | 0 | 0 | 0 | 13 | 0 | 0 | 0 | 0 | 0 | 0 | 0 | 0 | 0 | 523 | 5 | 6 | 2 | 14 |
| 1 | denovo350 | 98% | *Pseudomonas sp.* | *Gammaproteobacteria* | 0 | 0 | 0 | 0 | 0 | 39 | 0 | 0 | 0 | 0 | 0 | 0 | 0 | 0 | 0 | 0 | 0 | 0 | 0 | 0 |
| 3 | denovo248 | 99% | *Staphylococcus pasteuri* | Firmicutes | 0 | 0 | 0 | 0 | 0 | 52 | 0 | 0 | 1 | 0 | 0 | 38 | 0 | 0 | 0 | 0 | 2 | 2 | 0 | 0 |
| 6, 8 | denovo573 | 94-99% | *Arthrobacter sp.* | Actinobacteria | 0 | 0 | 0 | 0 | 0 | 0 | 0 | 0 | 0 | 17 | 0 | 0 | 0 | 0 | 0 | 0 | 0 | 0 | 0 | 0 |
| 7 | denovo65 | 99% | *Lentimonas marisflavi* | Verrucomicrobia | 0 | 0 | 24 | 0 | 0 | 0 | 0 | 0 | 0 | 15 | 0 | 0 | 0 | 0 | 0 | 564 | 26 | 23 | 81 | 149 |
